# Thermoset Shape Memory Polymer Variable Stiffness 4D Robotic Catheters

**DOI:** 10.1002/advs.202103277

**Published:** 2021-10-31

**Authors:** Michael Mattmann, Carmela De Marco, Francesco Briatico, Stefano Tagliabue, Aron Colusso, Xiang‐Zhong Chen, Jonas Lussi, Christophe Chautems, Salvador Pané, Bradley Nelson

**Affiliations:** ^1^ Institute of Robotics and Intelligent Systems ETH Zürich Tannenstrasse 3 Zurich CH‐8092 Switzerland; ^2^ Department of Chemistry Materials and Chemical Engineering Politecnico di Milano Milan 20131 Italy

**Keywords:** catheters, composite materials, shape memory polymers, thermoset polymers, variable stiffness

## Abstract

Variable stiffness catheters are typically composed of an encapsulated core. The core is usually composed of a low melting point alloy (LMPA) or a thermoplastic polymer (TP). In both cases, there is a need to encapsulate the core with an elastic material. This imposes a limit to the volume of variable stiffness (VS) material and limits miniaturization. This paper proposes a new approach that relies on the use of thermosetting materials. The variable stiffness catheter (VSC) proposed in this work eliminates the necessity for an encapsulation layer and is made of a unique biocompatible thermoset polymer with an embedded heating system. This significantly reduces the final diameter, improves manufacturability, and increases safety in the event of complications. The device can be scaled to sub‐millimeter dimensions, while maintaining a high stiffness change. In addition, integration into a magnetic actuation system allows for precise actuation of one or multiple tools.

## Graphical abstract

In this work, an advanced 4D robotic medical catheter based on a processable thermoset shape‐memory polymer composite, is reported. The developed electrically conductive composite allows for superior processability and further miniaturization maintaining high stiffness variations and fast transition speeds. The capabilities are demonstrated by performing robotic manipulations with multiple magnetic tools.

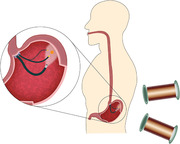

## 1 Introduction

**Figure 1:**
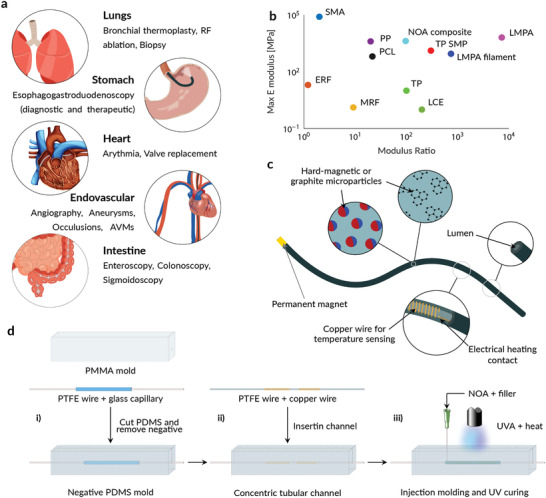
Design and manufacturing of the composite variable stiffness catheter. a) Possible applications of a VSC. The VSC can provide increased push‐ability in hard‐to‐reach areas of the body or increase the reachable workspace in open volumes. b) Stiffness properties of state‐of‐the‐art variable stiffness materials (SMA: shape memory alloy, ERF: electrorheological fluids, PP: piezoelectric polymer, PCL: polycaprolactone, MRF: magnetorheological fluid, TP SMP: thermoplastic shape memory polymer, TP: thermoplastic, LCE: liquid crystal elastomer, and LMPA: low melting point alloy). The maximum tensile elastic modulus versus moduli ratio (maximum over minimum) of reported and analyzed variable stiffness materials. Adapted from.^[^ ^21^ ^]^ c) Schematic illustration of the VSC: graphite or neodymium particles are embedded in the VS polymeric body. The lumen provides a working channel for tools or fluid injections, while the permanent magnet on the tip allows for magnetic actuation. The embedded copper wires allow for temperature and stiffness control. d) Fabrication method of the VSC, based on an injection molding process. The PDMS mold is fabricated i) with a PMMA structure, a PTFE wire, and a glass capillary. The mold is ii) filled with a copper wire, coiled around a PTFE wire and iii) NOA injected into the remaining space. UV–A light and heat are used to cure the thermoset material.

Soft continuum robots, such as catheters and endoscopes, have demonstrated their advantages in many medical applications (**Figure** **1**a) and have become a key component in minimally invasive medicine. These devices allow for safer therapeutic and diagnostic procedures by reducing the risk of infections and physical stress on the body, increasing the benefits and positive outcomes, and reducing recovery time.^[^ ^1^, ^2^, ^3^, ^4^, ^5^, ^6^ ^]^

Despite these advantages, reported tools currently face multiple challenges. Endovascular catheters, for example, have a predefined stiffness and are usually guided with the aid of an additional guidewire. To reach the targeted location, surgeons often require multiple tools increasing both the cost and time required for each intervention. In contrast, endoscopes and cardiac catheters are usually guided with the aid of pull wires. These allow deflection of the tip segment and variation of the tool stiffness. The increased tip dexterity is, however, limited to a single segment, which limits the reachable workspace. To reach all targeted areas, surgeons are still required to use additional introducer sheaths.

In order to solve these problems, substantial research has been invested in small 4D soft robots,^[^ ^7^, ^8^, ^9^, ^10^, ^11^, ^12^, ^13^ ^]^ which are robots that can change their shape and mechanical properties over time. An example of this is variable stiffness (VS) technologies, which enable the development of surgical tools capable of overcoming these limitations.

The compliance of these tools enables safe navigation with the ability to increase the stiffness when interaction with the tissue is required (e.g., puncturing, grasping, biopsy, etc.).^[^ ^14^ ^]^ Multiple VS segments also increase the reachable and dexterous workspace,^[^ ^15^ ^]^ reducing the number of tools required for surgical interventions.

Granular or layer jamming‐based tools have been investigated at larger scales (>5 mm) and have proven to be a valid option for endoscopic applications. The need for multiple layers or a discrete amount of particles,^[^ ^16^, ^17^, ^18^, ^19^ ^]^ does, however, require a trade‐off between stiffness change and scalability. However, VS tools based on thermally induced phase transitions can be miniaturized. Our group has recently developed a variable stiffness catheter (VSC) based on low‐melting point alloys (LMPAs) and controlled using external magnetic fields.^[^ ^15^ ^]^ The introduction of multiple VS sections allowed more complex shapes to be reached and a larger workspace to be covered when compared to standard soft tools. Low melting point alloys do, however, rely on toxic materials which, in the case of leakage, could cause permanent damage to the health of the patient.^[^ ^15^, ^20^ ^]^ VSC based on thermoplastic polymers is instead associated with a limited stiffness variation and maximum modulus (Figure 1b).^[^ ^21^, ^22^, ^23^, ^24^, ^25^ ^]^ Furthermore, these materials all rely on a phase transition between a rigid and a molten state and therefore require an encapsulation, which reduces the volume of VS material or increases the overall dimension.

Our variable stiffness catheter is based on a thermoset shape memory polymer (SMP) creating a magnetically steerable 4D robotic device.^[^ ^8^ ^]^ The device can be scaled to sub‐millimeter dimensions and exhibits a modulus change of more than two orders of magnitude. The device exploits the glass transition of thermoset shape memory polymers, thereby avoiding the molten state and the requirement for an encapsulation structure. The stiffness of the proposed tool can be precisely controlled by adjusting the material temperature. The soft robot can be easily manipulated in open volumes and covers a large workspace using magnetic actuation. The proposed technology increases the dexterity of magnetic tools, guarantees safety, and allows interaction with biological tissue. Additionally, multiple magnetic tools can be controlled within the same magnetic actuation system, as any number of tools can be locked in position while manipulating a selected individual tool. The introduction of 4D soft robotics extends the range of medical procedures that can be executed with minimally invasive approaches.

## 2 Results and Discussion

### 2.1 Design and Developement

We selected thermoset shape memory polymers due to their high modulus, the large modulus variation on glass transition, and their biocompatibility. As can be seen in Figure 1b, both thermoset SMPs and encapsulated LMPA structures maximize the stiffness ratio (Ratio  = *E*_stiff_ /*E*_flexible_) and exhibit high stiffness. Thermosetting polymers, in contrast to thermoplastic polymers that are used in VSCs, maintain a solid‐like behavior even after the glass transition temperature (*T*_g_). By moving to the glass transition, we were able to overcome the need for an encapsulation structure and maximize the volume of VS material.

NOA86H is a heat‐ and UV‐curable polyurethane based SMP. Its glass transition temperature ranges from 30 °C to 80 °C depending on the shape and the curing process. Below *T*_g_, the polymer is in a glassy state with a modulus of ≈3 GPa, while above *T*_g_, the polymer is in a rubbery state with a modulus of ≈3 MPa. The phase transition from glassy to rubbery state, the high stiffness ratio, the tunable transition temperature, and the USP class VI biocompatibility make NOA86H a good choice for a biocompatible VS tool based on a single VS material. We analyzed the compounding of thermally and electrically conductive powders in order to overcome the low thermal and electrical conductivity of SMPs^[^ ^26^ ^]^ when compared to LMPAs. Thermal conductivity is an important property as it determines the transition speed between the stiff and flexible configuration. A high conductivity decreases the thermal resistance and increases the transition speed. Electrical conductivity is also relevant, as a conductive material would allow the use of Joule heating instead of using an additional heating element. The simplified control circuit would therefore result in a more compact overall design. Figure 1c shows an illustration of the proposed VSC. The tool is composed of a cylindrical body made of NdFeB (5 *μ*m sized on average) or graphite particles embedded in a polyurethane polymeric matrix (NOA86H), an electric heating contact for each VS section, a temperature sensing coil for each section, and a permanent magnet.

**Figure 2:**
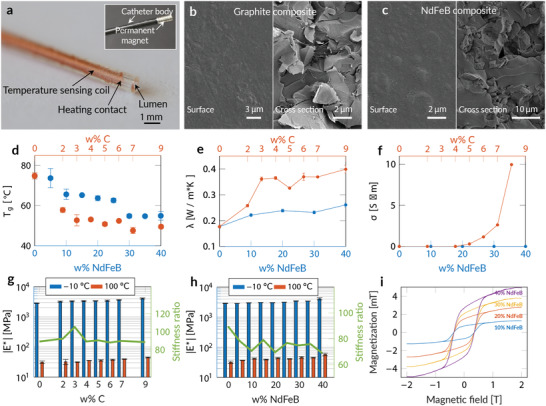
Characterization of the variable stiffness SMP composite. a) Variable stiffness catheter without filler showing the embedded control structure. The copper wire was uniformly coiled around the inner lumen over the entire length of the VS segment. b) SEM image of the catheter surface with embedded graphite and c) NdFeB particles. d) Glass transition temperature (*T*_g_) versus concentration of NdFeB and graphite. e) Thermal conductivity of NdFeB and graphite in a polymeric matrix. f) Electrical conductivity of NdFeB and graphite in a polymeric matrix. Stiffness ratio and complex modulus values in the rubbery and glassy state versus g) increasing concentration of graphite and h) NdFeB. i) Hysteresis curves of NdFeB in a polymeric matrix.

The catheters were fabricated by an injection molding process (Figure 1d). First, a transparent mold was made from silicone (Dow, Sylgard 184) using a negative template made by a glass capillary and a Teflon (PTFE) filament (Figure 1d‐i). Then, the upper part of the mold was cut and the negative removed. An additional Teflon filament, used as negative for the catheter lumen and as a centering element for the heating and control circuit, was inserted into the channel (Figure 1d‐ii), and the SMP composite was injected. The catheter surface was cured with UV‐A light, and the bulk material was cured in an oven at 120 °C (Figure 1d‐iii). Finally, the VS tool was obtained by removing the PTFE wire and gluing the permanent magnet onto the tip. An example without any filler, to observe the internal structure, is shown in **Figure** **2**a. The main body has an outer diameter of 1.3 mm and an inner lumen with diameter of 0.4 mm. The temperature measurement was achieved using an enameled copper wire coiled around the inner lumen of the tool and relies on the resistivity change with a temperature change. The sensing coils were characterized by the mean of the thermal coefficient *α*. The resistance variation over temperature was measured and the thermal coefficient computed with the linear approximation *ρ* (*T*) = *ρ*_0_ (1 + *α*(*T* − *T*_0_)) with *ρ* the resistivity and *ρ*_0_ the resistivity at *T*_0_ (Figure S6, Supporting Information). Heating was achieved with two electrical contacts placed at the extremities of each VS section. When a voltage was applied, heat was generated by Joule heating in the conductive VS material.

Figure 2b,c shows SEM images of the cross‐section of graphite and NdFeB filled cylinders. We can observe a uniform particle distribution across the catheter cross‐section and the absence of particles on the catheter surface, which can be attributed to surface tension or an exclusion effect of fluid dynamic nature. Figure 2d shows the *T*_g_ as a function of filler material and concentration. We observed an approximately linear decrease in *T*_g_with increasing concentration of both graphite and NdFeB. The fillers seem to inhibit the formation of netpoints, so that the degree of crosslinking decreases at increasing filler content. The effect is an increase in filled polymer mobility which is reflected by the *T*_g_decrease. We were able to reduce the transition temperature to a temperature compatible with the devised application of ≈50 °C with both fillers. In Figure 2e, we observe the effect of fillers on the thermal conductivity of the composite material. The thermal conductivity increases rapidly with the addition of graphite and reaches a plateau after a concentration of 3 wt%, whereas the addition of NdFeB results in a slower linear increase. Graphite doubles the thermal conductivity and the heat flux with a given temperature gradient and influences the electrical conductivity of the composite (Figure 2f). We were able to observe an exponential conductivity increase of up to 1 Sm with the addition of 9 wt% of graphite. In contrast, the addition of NdFeB did not have any influence on the electrical conductivity. The conductivity was negligible (*σ* ≈ 0 *S* *m*) across all concentrations. While observing a conductivity increase in the bulk material, we were able to observe superior electrical insulation of the catheter surface. The measured single point resistance exceeded the range of our measurement setup (1 GΩ). This behavior can be explained by the absence of conductive particles on the catheter surface.

The influence of filler material and concentration on the mechanical properties (Figure 2g,h) was characterized with the absolute value of the complex modulus E*, defined according to Equation (1), with *E*′ being the storage modulus and *E*′′the loss modulus.

(1) $$|E^{*}|\;=\sqrt{{E^{\prime }}^{2}+{E^{\prime \prime }}^{2}}$$

We observed an increase of modulus with increasing filler concentration both in the glassy and the rubbery state. The addition of up to 9 wt% of graphite resulted in a linear increase of up to 1.5 times in the glassy and rubbery state (Figure 2g), reaching a maximum modulus of $|E_{C,\;\text{glassy}}^{*}|\;=\;4.1\;\mathrm{GPa}$. The addition of neodymium resulted in a similar increase in the glassy state reaching a maximum modulus of $|E_{\mathrm{NdFeB},\;\text{glassy}}^{*}|\;=\;4.05\;\mathrm{GPa}$ (Figure 2h). We observed a higher modulus increase in the rubbery state reaching a value of two times higher than without filler. This resulted in a decrease in modulus ratio with increasing filler concentrations (ratio = 70). In comparison to NdFeB, we observed a stable modulus ratio with the addition of graphite (ratio = 93).

In order to assess the magnetic steerability, we analyzed the magnetic properties of the NdFeB filled structures and their response to external magnetic fields. The saturation magnetization of the samples (Figure 2i) increased linearly with filler concentration, as expected from the analytical formulation of the magnetization *M*_S_ = *M*_Sp_  · *φ*, with *M*_Sp_ being the saturation magnetization of the particles and *φ* the volume fraction magnetic particles.

With the addition of graphite, we were able to fabricate a thermoset SMP based VS tool with superior mechanical, electrical, and thermal properties. The addition of NdFeB improved the thermal and magnetic properties. However, this resulted in a decrease of modulus ratio, and the resulting magnetization was not sufficient for magnetic steering (Figure S3, Supporting Information) without an additional permanent magnet.

**Figure 3:**
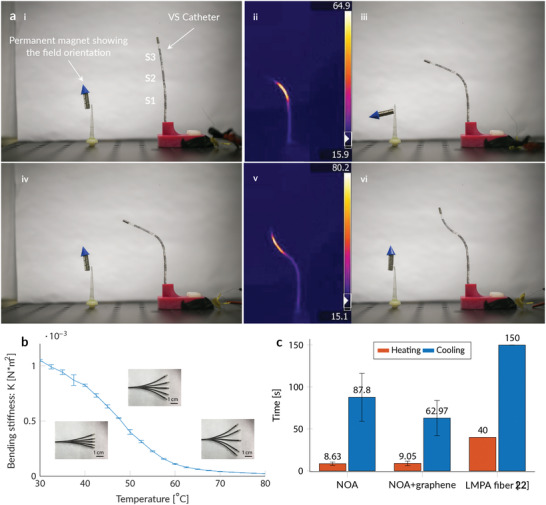
Characterization of the variable stiffness catheter. a‐i) Magnetic actuation of a three section VSC allows complex configurations to be reached in an open volume. ii,iii) When S2 was heated, the segment could be easily deformed. In a second step, iv) S2 was locked in position, and v,vi) S3 was heated and deflected. b) Stiffness of a segment as a function of temperature. The stiffness can be accurately controlled allowing for a controlled deflection limitation. c) Transition time as a function of material: the transition speed can be increased with the addition of thermally conductive powders.

The improvements to the VS material allowed us to demonstrate the capabilities of our VSC. **Figure** **3**a shows the experimental validation of a multi‐section VS catheter, demonstrating our ability to locally change the tool stiffness and reach a shape that would not be possible with standard magnetic tools. The prototype was fabricated without any filler (Figure 3a‐i), was heated with an additional heating wire, and has three VS sections (S1, S2, and S3). When the heated segment (S2) reaches the glass transition temperature (Figure 3a‐ii), it can be bent by ≈90° with a field of 40 mT, while the remaining segments can maintain their shape (Figure 3a‐iii). When cooled to room temperature (RT), the deformed segment maintained its deformed configuration upon the application of an 80 mT magnetic field rotated by 90° (Figure 3a‐iv). The proposed tool can withstand a torque *T* of 6.5 Nmm applied to the catheter tip. The applied torque can be calculated according to the analytical description *T*  =  *M* × *B*, where *M* denotes the magnetic moment of the permanent magnet and *B* the applied magnetic field. When a second section (S3) was heated (Figure 3a‐v), the section bent in order to align itself to the applied field (Figure 3a‐vi).

The bending stiffness of the VSC, defined as *K* = E*I in, which *E* is the Young's modulus and *I* the moment of inertia, can be accurately controlled by adjusting the temperature of the SMP. Figure 3b displays the measured mean bending stiffness while adjusting the temperature of the VSC. We performed three measurements for each temperature set point during both the heating and cooling processes. We observed highly stable and repeatable stiffness control resulting in little to no deviation between the different measurements. Additionally, we observe that a temperature limit at 60 °C would only marginally affect the stiffness variation of the device. The high surface temperature can be additionally decreased with an additional insulation layer (Figure S7, Supporting Information). We have shown that a silicone layer of 0.15 mm thickness allows to decrease the surface temperature by more than 15%. The additional layer has only marginal effect on the dimension and stiffness while allowing a safe surface temperature below 50 °C.

The improved thermal properties of the graphite‐filled structures allowed the cooling speed to be to drastically reduced, while maintaining a similar heating performance. Figure 3c shows that the addition of 7wt% of graphite allowed the heating speed to be reduced by a third, while maintaining a heating time of ≈7 s. Due to the reduced thermal resistance and increased heat flow to the surroundings, a heating power increase of ≈35% was required to maintain the heating speed. Compared with the LMPA filaments reported by Tonazzini et. al.,^[^ ^27^ ^]^ the SMP structures provided better cooling and heating performances.

We attribute the improved properties to the absence of an encapsulation and insulation layer.

The increased transition speed reduces the intervention time by reducing the waiting time during the stiffness transition. The proposed VSC allows the user to soften and steer the device within 9s while the shape can be fixed within ≈60s in air.

Our design allows miniaturization, as the catheter can be heated above its transition temperature by direct Joule heating (Figure S4b, Supporting Information) with a current of only 5 mA, and without an embedded heating structure. Additionally, it increases procedure safety and reduces energy consumption. Finally, the increased fluoroscopic contrast of NdFeB composites (Figure S4a, Supporting Information) enhances localization and steering in the human body, further increasing the safety of the intervention.

### 2.2 Applications

**Figure 4:**
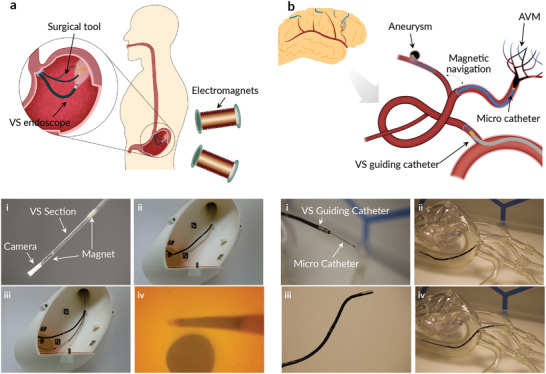
Applications of a composite variable stiffness catheter. a) Schematic illustration of an endoscopic procedure performed with a VS endoscope. The VS endoscope is i) magnetically steered and locked in position when the desired configuration is reached. This allows for the ii) insertion and magnetic actuation of the second tool. iii) The second tool is used to perform the desired intervention with a iv) visual feedback given from the endoscopic camera. b) Illustration of an endovascular application of the VS technology. i) A VS guiding catheter in combination with a magnetic micro catheter can be used for the treatment of endovascular diseases. ii) A VS guiding catheter can be used, in the soft state, to reach the desired location and be locked in position to ease the insertion of a micro catheter. iii) A VS micro catheter can be navigated to the targeted position (e.g., aneurysm) for the iv) injection of an embolization agent.

By adding functional elements, we were able to demonstrate additional capabilities and applications of the thermoset SMP‐based variable stiffness catheter. The stiffness variation allows a targeted configuration to be reached in the rubbery state and the shape to be fixed after cooling. This could be used for medical interventions where multiple magnetic tools need to be manipulated within the workspace of an electromagnetic navigation system (eMNS) (Movie S2, Supporting Information). For example, in endoscopies (e.g., natural orifice translumenal endoscopic surgery), camera is often used to image the procedure, and a second tool is used to perform the therapeutic intervention (**Figure** **4**a). The proposed endoscopic tool is composed of four VS sections with a length of 35 mm, four permanent magnets, a camera, and a light source (Figure S5b, Supporting Information). Each VS section is independently controllable and enables high dexterity in open volumes. Figure 4a (i‐iv) shows a possible workflow for such an intervention. The tool can be magnetically steered to the desired location (Figure 4a‐ii) and subsequently locked in position for the insertion and magnetic actuation of the second tool (Figure 4a‐iii). The second tool can then be manipulated to perform the desired intervention with a visual feedback provided by the endoscopic camera (Figure 4a‐iv). Finally, both tools can then be removed.

The low transition temperature and high stiffness range make this VSC a viable candidate for endovascular procedures. Endovascular catheters are currently limited to specific applications and the length, stiffness, and geometry are optimized for given anatomies and applications. Guiding catheters, for example, exist in over 200 configurations, requiring selecting one guide catheter at the beginning of the procedure and increasing the procedure time and cost if the guiding catheter needs to be changed during the intervention. A VS guiding catheter could overcome these limitations as it can be navigated to the targeted vessel and locked in position to sustain and ease the introduction of the micro catheter (Figure 4b). Such a catheter would allow a stiffness variation based on the actual conditions and requirements, allowing the navigation of complex anatomies in the flexible state while sustaining complex shapes in the rigid state. The demonstrated VS guiding catheter has an outer dimension of 3 mm and a lumen with an ID of 1.65 mm. The catheter is composed of one VS section with a length of 100 mm and two permanent magnets (Figure S5a, Supporting Information). The magnetic micro catheter has an outer diameter of 0.8 mm and a lumen with an ID of 0.4 mm (Figure 4b‐i).

A possible workflow is illustrated in Figure 4b (ii to iv). The guiding catheter can be inserted and magnetically manipulated in order to reach the targeted location (Figure 4b‐ii) and can subsequently be locked in position giving a structural support for the insertion of the microcatheter (Figure 4b‐iii). The magnetic microcatheter can be inserted (Figure 4b‐iv) and navigated to the desired position with the aid of magnetic manipulation. Once the targeted location is reached, the targeted intervention can be performed. Finally, both tools are retracted.

## 3 Conclusion

Our VS magnetic device provides a significant increase in procedure safety while maintaining a high modulus ratio and transition speed. Its biocompatible material and the transition from a glassy to a rubbery state provide additional safety in the event of complications. The VS magnetic device can be selectively softened for increased safety and steering, or stiffened for improved stability and force transmission. The use of composite materials provides the potential for further miniaturization and allows for faster stiffness switching. The use of VS devices opens up a broader range of applications for minimally invasive interventions. Additionally, the use of radiopaque particles enables visualization in a clinical setting and further increases the procedure safety. The surface temperature, of up to 50 °C, can be further decreased with an optimized insulation material or by further optimization of the glass transition.

## 4 Experimental Section

### Mechanical Characterization

In order to measure the mechanical properties and glass transition temperature, cylindrical samples were characterized in tension by dynamic mechanical analysis using a Dynamic Mechanical Analyzer RSA3 (TA Instruments). This technique relies on the application of a sinusoidal strain to which the material responds with a sinusoidal, out of phase, stress. The output signal can be deconvolved in an in‐phase or in‐quadrature signal. From this analysis, the conservative component of complex modulus, or storage modulus, *E*′, and the dissipative component of the complex modulus, or loss modulus, *E*′′ can be determined along with the complex modulus E*, its absolute value |E*|, and the loss factor tan(*δ*).

(2) $$E^{*}=E^{\prime }\;+iE^{\prime \prime }\;\;\;\;\left|E^{*}\right|=\sqrt{{E^{\prime }}^{2}+{E^{\prime \prime }}^{2}}\;\;\;\;\;\tan\left(\delta \right)=\frac{E^{\prime \prime }}{E^{\prime }}$$

Each cylindrical sample, with an outer diameter of 1.5 mm and a clamping length of 15 mm, was subjected to a sinusoidal strain of 0.1% and a frequency of 1 Hz.

Two temperature ramps were performed from −10 °C to 120 °C at a heating rate of 2 °C min^−1^. After the first ramp, the sample was quenched to −10 °C at the cooling rate of −50 °C min^−1^.

By comparing the two scans, we could verify the complete crosslinking of the thermoset resin after cylinder production. From the temperature ramps, a characteristic value for |E*| in the glassy state was determined at −10 °C, and that of |E*| in the rubbery state was determined at 100 °C, which is well above the glass transition temperature. The latter was determined as the temperature corresponding to the tan(*δ*) peak.

### Thermal Characterization

In order to quantify the effect of fillers on the thermal conductivity, cubic samples were analyzed in a steady state with a guarded hot plate setup. The sample was placed between a hot plate and a cold plate. The temperature of the hot plate was heated to 40 °C, while the cold plate was cooled to 15 °C. The temperature difference between the two sample surfaces (Δ*T*), as well as the heat flow *q*, was measured with a heat flux sensor (PHFS‐01, FluxTeq). The thermal conductivity could be calculated using

(3) $$\lambda \;=\frac{q^{\prime \prime }\cdot d}{\Delta T}\;\;\mathrm{with}\;q^{\prime \prime }=\frac{q}{A}$$

### Electrical Characterization

To quantify the electrical properties of the filled NOA samples, the electrical resistance of cylindrical samples was measured with a Picoammeter (6487 Picoammeter / Voltage source, Keithley). Each cylindrical sample, with an outer diameter of 1.5 mm and a length of 50 mm, was subjected to a voltage ranging from 0.001 to 10 V and the electrical resistance computed with the measured current.

(4) $$\sigma \;=\frac{L}{R\cdot A}$$

### Magnetic Characterization

To assess the magnetic properties of the NdFeB filled samples, cylindrical samples were analyzed with a vibrating sample oscillometer (FCM‐10, MicroSense). Samples with a diameter of 1.3 mm and a length of 1 cm were subjected to a magnetic field ranging from −2 to 2 T, while the magnetization was measured. Magnetized cylindrical samples with a diameter of 1.3 mm and a length of 5 cm were fixed in an electromagnetic navigation system (CardioMag) and subjected to a magnetic field of 140 mT perpendicular to the sample axis. The resulting deflection was measured with an image processing approach.

### Bending Stiffness Characterization

To assess the bending stiffness, the catheters were subjected to a three‐point bending test. The catheters were fixed on two supports at a distance of 20 mm, while a force of 5 mN was applied between the two supports. The force–deflection curve was measured with a micro force sensing probe (FT‐S10000, FemtoTools). The bending stiffness *K* could be calculated according to the following formula:

(5) $$K\;=\;E\cdot I\;=\frac{\partial P}{\partial s}\;\frac{L^{3}}{48}$$

where *L* is the length between the two supports. The bending stiffness was measured ten times for each temperature level.

### Cooling/Heating Speed

To assess the cooling and heating speed of the catheter, the authors analyzed the resistance of the measurement wires over time. The heating time was measured as the time required to reach a resistance level corresponding to the soft state. The cooling time was defined as the time required to recover a temperature of 30 °C starting from the flexible state. Both experiments were performed in air.

### Magnetic Manipulation

To assess the magnetic response and magnetically manipulate the VSC, the CardioMag eMNS had been used. The magnetic fields were generated by eight electromagnets arranged in the same manner as in the OctoMag eMNS.^[^ ^28^ ^]^ It generated magnetic fields up to 120 mT in a workspace of ≈30 cm × 30cm × 30 cm.

The generated magnetic field generated a magnetic torque on the permanent magnet of the VSC according to *T*  =  *M* × *B*, where *M* denotes the magnetic moment of the permanent magnet and *B* the applied magnetic field. The permanent magnet thus wanted to align with the applied magnetic field.

### Stiffness Control

In order to control the stiffness of the VSC, a PID controller had been implemented. The controller took the measured resistance of the sensing coil and output an adequate heating voltage.

## Conflict of Interest

The authors declare no conflict of interest.

## Supporting information

Supporting Information

Supplemental Movie 1

Supplemental Movie 2

## Data Availability

Research data are not shared.
