# Supplementary material for: Thermoset Shape Memory Polymer Variable Stiffness 4D Robotic Catheters
Source: Adv Sci (Weinh). 2021 Oct 31;9(1):2103277. doi: 10.1002/advs.202103277 (PMC8728812; doi:10.1002/advs.202103277)
Supplement: Supplementary file 1 — Supporting Information [file ADVS-9-2103277-s003.pdf]

WILEY-VCH

© 2021 Wiley-VCH GmbH

ADVANCED SCIENCE
Open Access

Supporting Information

for *Adv. Sci.,* DOI: 10.1002/advs.202103277

Thermosetting Shape Memory Polymer-Based Composite Variable Stiffness Catheters

Michael Mattmann, Dr. Carmela De Marco, Dr. Francesco Briatico, Stefano Tagliabue, Aron Colusso, Dr. Xiang-Zhong Chen, Jonas Lussi, Dr. Christophe Chautems, Prof. Salvador Pané, Prof. Bradley Nelson*

Fabrication of the Variable stiffness catheter:

The variable stiffness catheters (VSC) were fabricated using commercially available thermoset shape memory adhesives (NOA86H, Norland Products) as the variable stiffness material and NdFeB (MQFP-15-7-20065-089, Magnequench) or graphite powders as the composite materials. Alternative variable stiffness materials and fillers could be used, depending on the desired material properties.

The prototypes were fabricated by an injection molding process. The mold was made of silicone (Sylgard 184, Dow) using a negative template made by a glass capillary (OD 1.3 mm) and a Teflon (PTFE) filament (OD 0.4 mm). The silicon was cured in an oven at 120 °C for 20 min. Once cured, a cut in the upper part was performed in order to remove the negative template and insert the control circuit with the negative lumen. An enameled copper wire (OD 50 μm) was

coiled around a PTFE filament, acting as negative template for the catheter lumen and as a temperature sensor. The temperature measurement relies on the resistivity change of the embedded copper wire. Additional electrical contacts, achieved by enameled copper wires, were glued onto the PTFE filament and used for Joule heating of the VSC. The prepared assembly was placed concentrically in the silicon channel and the VS polymer injected through a syringe needle. The elasticity of the silicon mold sealed the cut and generated a tubular mold structure. The filled mold was initially subjected to UV-A light (315-400 nm) with an intensity of 6 W for 1 hour and subsequently heated to 120 °C for 2 hours in an oven. Finally, the cured VS structure was removed from the mold, the negative lumen extracted, and the permanent magnet glued on the catheter tip.

In this work, we tested different filler materials and concentrations with the goal to improve the overall material properties required for a magnetic VSC. Our goal was to analyze the effect on the stiffness variation, the control performance, the transitions speed, the magnetic steerability, the transition temperature, and the fluoroscopic contrast.

Description for the graphite and neodymium nanoparticle:

For the carbonaceous filler, a graphitic material available in the laboratory was used. In order to obtain information concerning the structure of the graphitic material, a Raman analysis (excitation wavelength 632 nm, laser power 5 mW) was carried out and SEM images taken.

Four spectra were recorded focusing on different regions of a powder sample deposited on a microscope slide (Figure S1a). The spectra show a very similar spectral pattern, suggesting that the sample is quite homogeneous. The common Raman pattern characterized by a strong G line and a weaker D band (Figure S1b) corresponds to that of a graphite-like material. The second order 2D Raman feature has the typical shape of a stack of graphene layers, suggesting that the crystalline structure in the c axis direction is partially preserved.

A direct comparison with a spectrum of a slightly disordered microcrystalline graphite, shows a remarkable similarity. On this basis, the material could be classified as a partially exfoliated micro-graphite. SEM images were recorded focusing on different regions of the powder sample deposited on an SEM sample holder (Figure S2a). The images show a particle size in the μm range.

For a Neodymium filler, an NdFeB powder available in the laboratory was used. The hard magnetic material is an isotropic metal powder (MQFP-15-7-20065-089) supplied by Magnequench. It is based on a neodymium-iron-boron (Nd-Fe-B) alloy with a median particle size of 5 μm (Figure S2b).

VS endoscope and VS guiding catheter:

The design of the VS endoscope is illustrated in Figure S5b. The design relies on four VS sections, four permanent magnets, a camera, and a light source. The four sections have a length of 35 mm, an OD of 2.5 mm, and an ID of 1 mm. All sections have independent heating and control connections that enable an independent stiffness control. Four magnets were positioned in between the different sections and at the tip. The camera on the tip was supplied by Misumi. The internal lumen was used to feed the camera and LED power cables.

The design of the VS guiding catheter is illustrated in Figure S5a. The catheter consists of one VS sections and two permanent magnets. The design relies on a dimension of 3 mm and a lumen of 1.65 mm. The lumen acts as channel for the microcatheter. The design does not include additional functional elements.

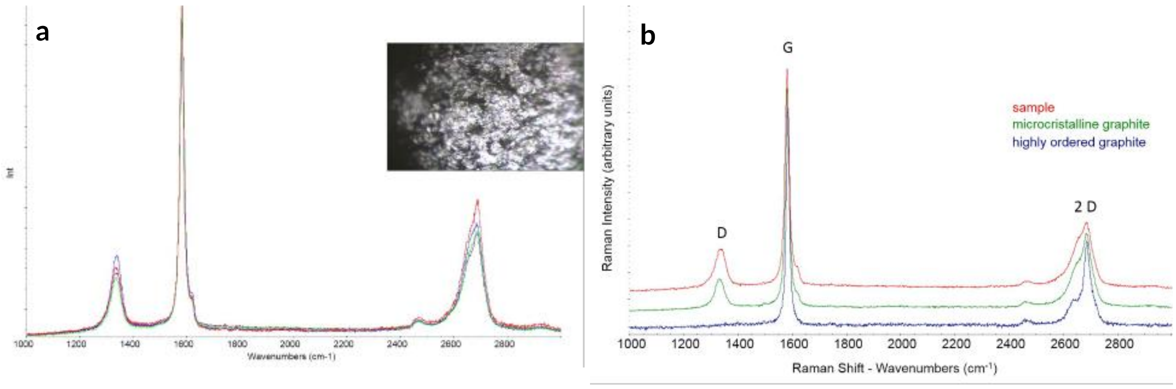

Figure S1. (a) Raman spectra corresponding to different sampling regions; spectra are recorded with $\lambda_{exc} = 632$ nm. (b) Comparison among different samples: available graphitic material (red); highly ordered graphite (blue); disordered graphite (green).

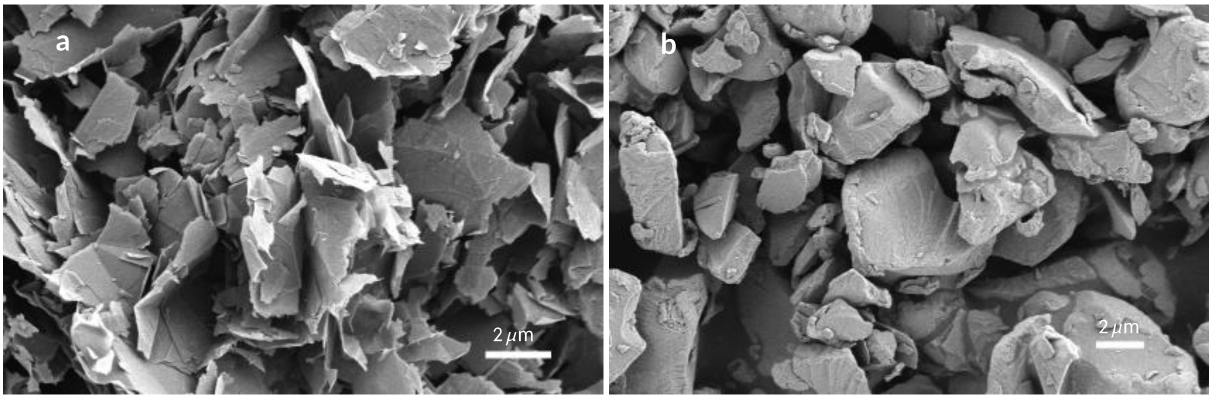

Figure S2. (a) SEM image of the graphite microparticles. (b) SEM image of the NdFeB microparticles.

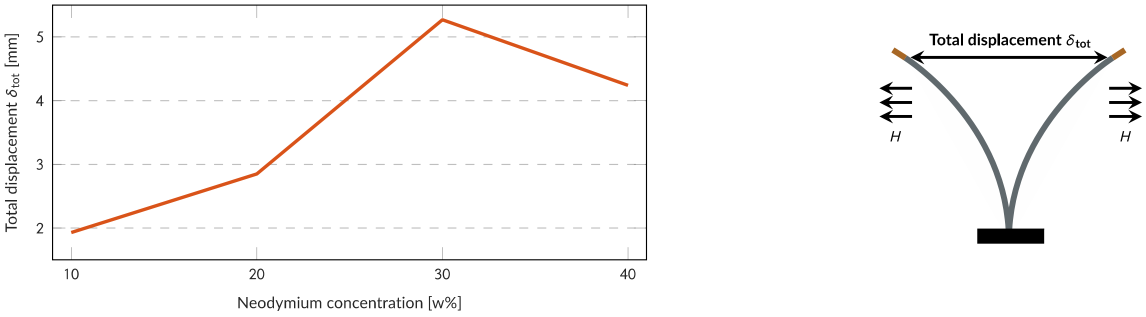

Figure S3. (right) Schematic illustration of the oscillation experiments. The proximal end of the 50 mm long catheter is fixed while the tip is free to deflect upon external forces. A magnetic field of 140 mT was applied and rotated from -90 deg to +90 deg with respect to the catheter main axis. (left) The observed deflection is shown as a function of NdFeB concentration.

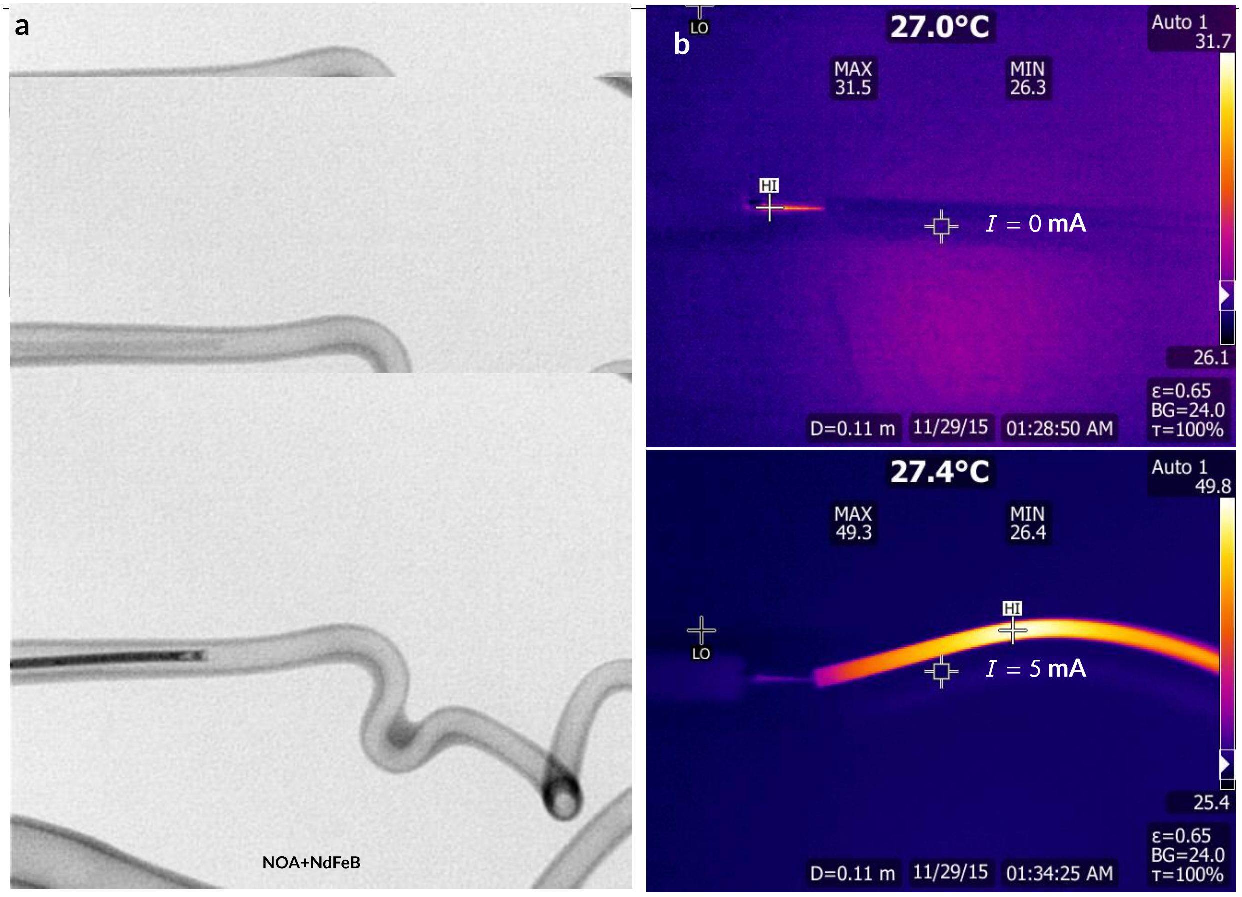

Figure S4. (a) Fluoroscopic images of NOA and NOA composites. Fluoroscopic images were recorded with a Ziehm Vision FD c-arm. NdFeB composite structures showed an increase in fluoroscopic contrast while graphite composites showed only a minimal influence. (b) Thermal image of graphite filled samples with a current flow of 0 mA (top) and 5 mA (bottom). A current of only 5 mA was enough to heat the VSC to a temperature of 50 °C using Joule heating.

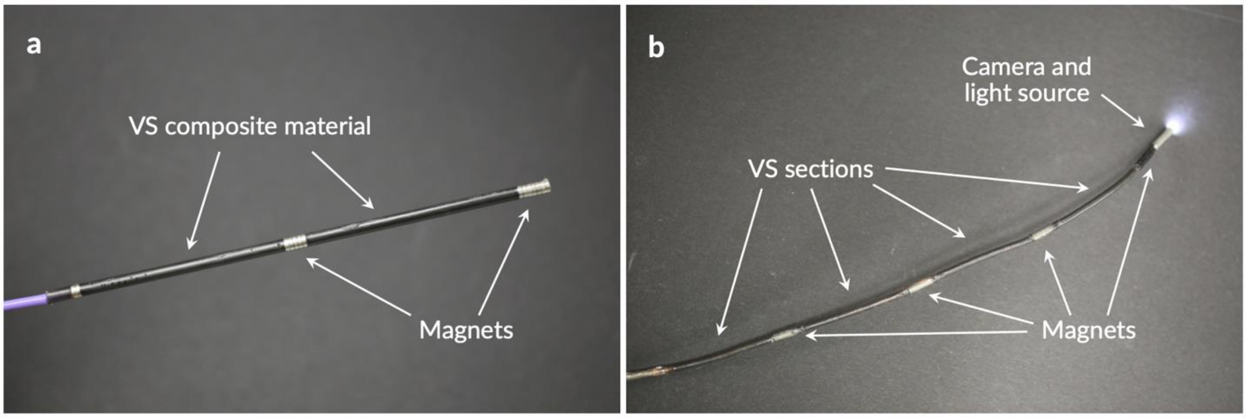

Figure S5. Variable stiffness tools. (a) A VS guiding catheter composed of a single variable stiffness section and two permanent magnetic sections. The VS guiding catheter has an outer diameter of 3 mm and an inner diameter of 1.65mm. The VS guiding catheter is compatible with all catheters having an outer diameter smaller than 1.6 mm. (b) A VS endoscope composed of four variable stiffness sections and four permanent magnets. The VS tool is equipped with a

camera and light source on the tip. The VS endoscope can be used in combination with other magnetic or manual tools and can provide visual feedback.

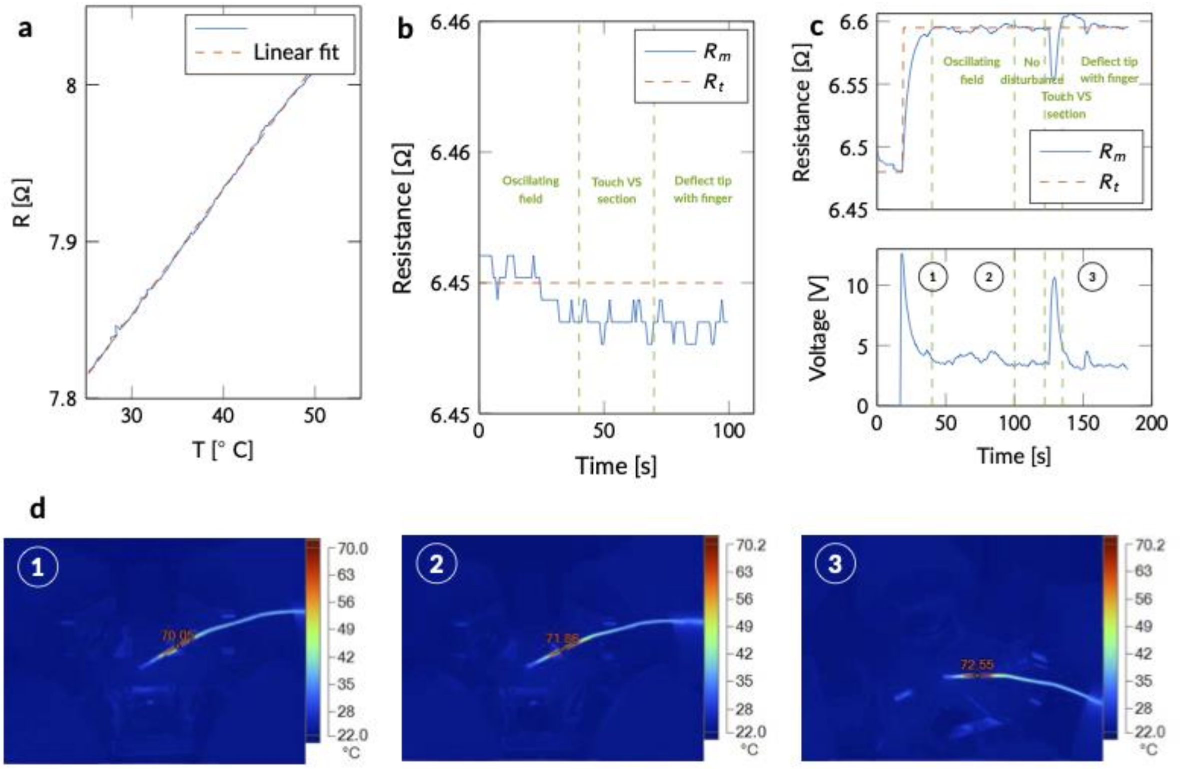

Figure S6. Uncontrolled and controlled resistance signal. (a) Calibration curve of the embedded sensing coil. The linear relation is used to compute the thermal coefficient of the system. (b) Uncontrolled resistance signal in the rigid state. Disturbances, such as oscillating fields, deflection, and touch have negligible effect on the control signal. (c) Controlled resistance signal with controller output. Magnetic fields, touch, and deflection have only marginal effect on the resistance signal and can be handled by the implemented control system. (d) IR images of the catheter at three different times. The constant surface temperature proves the validity of the constant resistance signal in (c).

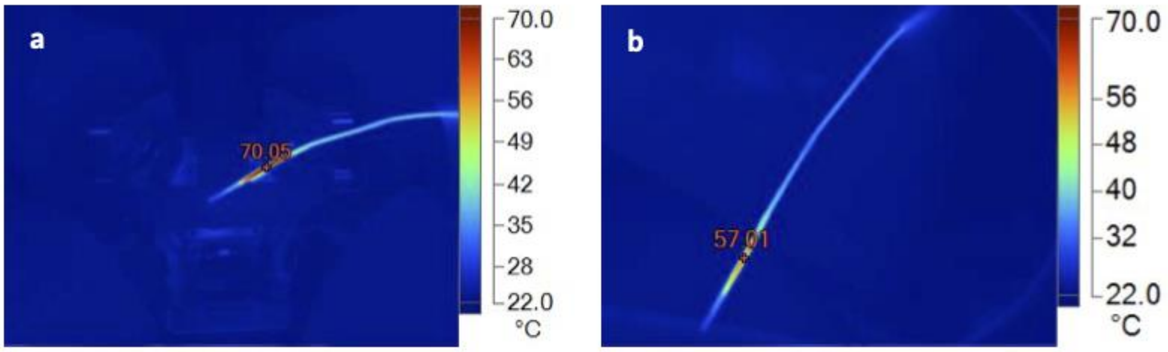

Figure S7. Influence of thermal insulation layer. Thermal images of uninsulated (a) and insulated catheter (b). The silicon insulation layer with a wall thickness of 0.15 mm allows to decrease the surface temperature by more than 15%.

Supplementary movies:
Movie S1: VS guiding catheter
Movie S2: VS endoscope
